# Alcohol drinking and risk of Parkinson's disease: a case-control study in Japan

**DOI:** 10.1186/1471-2377-10-111

**Published:** 2010-11-05

**Authors:** Wakaba Fukushima, Yoshihiro Miyake, Keiko Tanaka, Satoshi Sasaki, Chikako Kiyohara, Yoshio Tsuboi, Tatsuo Yamada, Tomoko Oeda, Takami Miki, Nobutoshi Kawamura, Nobutaka Sakae, Hidenao Fukuyama, Yoshio Hirota, Masaki Nagai

**Affiliations:** 1 Department of Public Health, Osaka City University Graduate School of Medicine, Osaka, Japan; 2 Department of Public Health, Faculty of Medicine, Fukuoka University, Fukuoka, Japan; 3 Department of Social and Preventive Epidemiology, School of Public Health, The University of Tokyo, Tokyo, Japan; 4 Department of Preventive Medicine, Graduate School of Medical Sciences, Kyushu University, Fukuoka, Japan; 5 Department of Neurology, Faculty of Medicine, Fukuoka University, Fukuoka, Japan; 6 Clinical Research Institute and Department of Neurology, Utano National Hospital, Kyoto, Japan; 7 Department of Geriatrics and Neurology, Osaka City University Graduate School of Medicine, Osaka, Japan; 8 Department of Neurology, Neurological Institute, Graduate School of Medical Sciences, Kyushu University, Fukuoka, Japan; 9 Human Brain Research Center, Kyoto University Graduate School of Medicine, Kyoto, Japan; 10 Department of Public Health, Saitama Medical University Faculty of Medicine, Saitama, Japan; 11 Other members of the Study Group are listed in the Appendix

## Abstract

**Background:**

Although some epidemiologic studies found inverse associations between alcohol drinking and Parkinson's disease (PD), the majority of studies found no such significant associations. Additionally, there is only limited research into the possible interactions of alcohol intake with aldehyde dehydrogenase (ALDH) 2 activity with respect to PD risk. We examined the relationship between alcohol intake and PD among Japanese subjects using data from a case-control study.

**Methods:**

From 214 cases within 6 years of PD onset and 327 controls without neurodegenerative disease, we collected information on "peak", as opposed to average, alcohol drinking frequency and peak drinking amounts during a subject's lifetime. Alcohol flushing status was evaluated via questions, as a means of detecting inactive ALHD2. The multivariate model included adjustments for sex, age, region of residence, smoking, years of education, body mass index, alcohol flushing status, presence of selected medication histories, and several dietary factors.

**Results:**

Alcohol intake during peak drinking periods, regardless of frequency or amount, was not associated with PD. However, when we assessed daily ethanol intake separately for each type of alcohol, only Japanese sake (rice wine) was significantly associated with PD (adjusted odds ratio of ≥66.0 g ethanol per day: 3.39, 95% confidence interval: 1.10-11.0, *P *for trend = 0.001). There was no significant interaction of alcohol intake with flushing status in relation to PD risk.

**Conclusions:**

We did not find significant associations between alcohol intake and PD, except for the daily amount of Japanese sake. Effect modifications by alcohol flushing status were not observed.

## Background

While smoking has been consistently associated with a decreased risk of Parkinson's disease (PD), a relationship between alcohol intake and PD still remains controversial. Some epidemiological studies reported that alcohol drinking was inversely associated with PD [1-4]. Conversely, the vast majority of investigations failed to find significant associations between alcohol intake and PD [5-13]. In a meta-analysis comparing drinkers with non-drinkers, the pooled odds ratio (OR) for PD risk was 0.81 (95% confidence interval (CI): 0.70-0.92) or 0.73 (95% CI: 0.57-0.92) when 13 case-control studies or 4 cohort studies, respectively, were included [14].

Alcohol drinking status, including frequency and amount, can be influenced by activities of alcohol dehydrogenases (ADHs) and aldehyde dehydrogenases (ALDHs). ALDH2, a key enzyme in the elimination of acetaldehyde, has a polymorphism that is prevalent in East Asians, but rare in Caucasians or Africans [15]. Those with inactive ALDH2 are likely to experience facial flushing responses due to acetaldehydemia after drinking alcohol, resulting in reduced alcohol intake with respect to both frequency and amount [16-19]. It has been reported that those with flushing responses drank significantly less hard liquor than those without [19]. Because aldehydes may react with dopamine both *in vitro *and *in vivo *[20,21], it might be hypothesized that relations between alcohol and PD risk vary according to flushing status. To our knowledge, only one study, a case-control study from Japan, investigated a possible effect modification by ALDH2 activity and found that average alcohol consumption was significantly lower among cases than controls regardless of ALDH2 genotype [3].

In the present study, we examined the association between alcohol drinking and PD risk using data from a hospital-based case-control study among Japanese. We also investigated whether flushing status, as a proxy variable of ALDH2 activity, modified the association.

## Methods

### Study subjects

Methodologies used in this study have been described previously [22,23]. Cases were recruited at 11 collaborating hospitals in Japan: 1 national and 3 university hospitals in Fukuoka, the largest prefecture in Kyushu Island in southern Japan, and 1 municipal, 3 university, and 3 national hospitals in Osaka, Kyoto, and Wakayama Prefectures, which are part of the Kinki region located in the midwestern part of Japan. Eligible cases were patients within 6 years of the onset of PD who had received treatment at one of the collaborating hospitals during the period from April 1, 2006 to March 31, 2008. The collaborating neurologists were responsible for the PD diagnoses, which were based on the UK PD Society Brain Bank clinical diagnostic criteria [24]. The neurologists in charge asked 298 eligible PD patients to take part: 250 were cooperative in answering the questionnaires and 48 declined (response rate: 84%).

Recruitment of control subjects was conducted at 3 of the same 11 hospitals: 1 university hospital in Fukuoka Prefecture, and 1 university and 1 national hospital in the Kinki region. Eligible control subjects were inpatients and outpatients without neurodegenerative diseases recruited from departments other than neurology (i.e., orthopedic surgery, ophthalmology, otorhinolaryngology, plastic surgery, and oral surgery) during the period from April 1, 2006 to March 31, 2008. Controls were not matched to cases either individually or by group. A total of 528 patients were approached by their attending physicians or our research nurses for recruitment as controls; 372 agreed and 156 declined (response rate: 70%). The study protocol was approved by the ethics committees of the 11 collaborating hospitals and written consent was obtained from all subjects.

### Information collection

Cases and controls filled out a set of 2 self-administered questionnaires and mailed them to the data management center or handed them to research nurses. Research technicians completed missing or corrected illogical data by telephone or direct interview. One questionnaire was used to collect information on "peak", as opposed to average, alcohol drinking status during a subject's lifetime. Subjects were asked to provide information on their drinking habits during the period in which alcohol consumption was the highest. We assessed drinking frequency, as well as volume of alcohol intake according to beverage types. The volume was subsequently converted to grams of ethanol, and values for each beverage type were added. The ethanol contents for calculation were as follows: 4.5% for beer, 15.5% for Japanese sake (rice wine), 25% for shochu (a distilled alcoholic beverage made in Japan), 12% for wine, and 43% for whisky. Both the daily and the weekly amounts of alcohol intake during the peak drinking period (for cases, only time prior to onset of PD) were estimated for each subject. The questionnaire also elicited information on several factors such as sex, age, smoking, education, and presence of medication history for hypertension, hypercholesterolemia, and diabetes. Although its validity has not been investigated, the questionnaire for this study was developed based on a comprehensive literature review of epidemiologic studies of factors associated with PD.

Alcohol flushing status was evaluated via questions as a proxy for detecting inactive ALHD2 [25], as follows: (a) Do you have tendency to flush in the face immediately after drinking a glass of beer (yes, no, or unknown)? (b) Did you have a tendency to flush in the face immediately after drinking a glass of beer during the first to second year after you started drinking (yes, no, or unknown)? Subjects who answered, "yes" to question (a) were classified as "current flushing", whereas those who answered "yes" to question (b) but not to question (a) were classified as "former flushing". The remaining subjects were classified as "never flushing". Among cancer-free Japanese men, when current or former flushing individuals were considered to have inactive ALDH2, the sensitivity and specificity of the questions were 90.1% and 88.0%, respectively [25].

Dietary habits during the preceding month were assessed using a self-administered, semi-quantitative, comprehensive diet history questionnaire (DHQ). Details of the DHQ's structure, calculation of dietary intake, and validity for commonly studied nutritional factors have been published elsewhere [26]. Dietary glycemic index, a measure of carbohydrate quality, not quantity, was calculated as described elsewhere [27]. In a validation study of 92 Japanese women and 92 Japanese men, Pearson's correlation coefficients between the DHQ and 16-day weighed dietary records were 0.43 and 0.38 for caffeine, 0.42 and 0.49 for cholesterol, 0.42 and 0.48 for vitamin E, 0.63 and 0.58 for vitamin B6, 0.68 and 0.52 for iron, and 0.50 and 0.58 for the dietary glycemic index, respectively [26]; [S Sasaki, unpublished observations, 2006]. Except for dietary glycemic index, dietary variables were energy-adjusted using the density method (amount per 4184 kJ of energy). Body weight and height were self-reported as part of the DHQ. Body mass index was calculated as weight (kg) divided by the square of height (m).

Because we failed to obtain information on peak alcohol drinking from 69 former drinkers (32 cases and 37 controls), they were eliminated from the analysis. Also excluded were 2 cases without available information on peak drinking prior to the onset of PD and 1 case and 4 controls whose information on any of the alcohol-related factors was missing. After further exclusion of 1 case and 4 controls due to missing data on other factors under study, 214 cases and 327 controls were subjected to final analysis.

### Statistical analysis

Frequency and amount of alcohol drinking were categorized into 3 levels based on the distribution of controls: 1) never drinkers, 2) current drinkers who consumed alcohol <6 days per week for frequency, 0.1-65.9 grams ethanol per day for daily amounts, and 0.1-219.3 grams per week for weekly amounts, and 3) current drinkers who consumed alcohol ≥6 days per week for frequency, ≥66.0 grams ethanol per day for daily amounts, and ≥219.4 grams per week for weekly amounts. Logistic regression analyses were employed to estimate ORs and 95% CIs of alcohol, using non-drinker as the reference category. The trend of association was assessed by assigning ordinal scores to the levels of independent variables.

The primary multivariate model included adjustments for sex, age, region of residence, pack-years of smoking, years of education, body mass index, alcohol flushing status, and presence of medication history (for hypertension, hypercholesterolemia, and diabetes) as potential confounders. Region of residence was classified into 2 categories (Fukuoka and Kinki); pack-years (1 pack/day for 1 yr) of smoking into 3 (none, 0.1-29.9, and ≥30.0); years of education into 3 (<10, 10-12, and ≥13 years); flushing status into 2 (never and former/current flushing); and presence of medication history into 2 (yes or no for each disease category). Age and body mass index were included as continuous variables. The fully adjusted model additionally incorporated several dietary factors including caffeine, cholesterol, vitamin E, vitamin B6, iron, and the dietary glycemic index as continuous variables. We also tested for statistical interactions of alcohol intake with flushing status by including product terms as independent variables in a fully adjusted model. All hypothesis testing was conducted assuming a 0.05 significance level and a 2-sided alternative hypothesis. SAS version 9.1 (SAS Institute, Inc., Cary, NC) was used throughout the analysis.

## Results

Thirty-five percent of the subjects were male and the mean age was approximately 70 years (Table 1). Compared with control subjects, cases were more likely to be older and thinner, report never having smoked, report never having medication histories for hypertension, hypercholesterolemia, or diabetes, and have a low intake of caffeine. There were no differences with respect to both the status of current drinking and alcohol flushing status, as well as sex, region of residence, education, and the other dietary variables.

**Table 1:** Characteristics of the study population

Variable	n (%) or mean (SD)		*P*-value
		
	Cases (N = 214)	Controls (N = 327)	
Sex (%)			0.86
Male	73 (34.1)	114 (34.9)	
Female	141 (65.9)	213 (65.1)	
Age (y)	67.9 (8.5)	66.4 (8.6)	0.04
Region of residence (%)			0.43
Fukuoka	81 (37.9)	135 (41.3)	
Kinki	133 (62.2)	192 (58.7)	
Pack-years of smoking (%)			0.007
None	164 (76.6)	211 (64.5)	
0.1-29.9	28 (13.1)	55 (16.8)	
≥ 30.0	22 (10.3)	61 (18.7)	
Education (%)			0.74
< 10 y	39 (18.2)	64 (19.6)	
10-12 y	110 (51.4)	157 (48.0)	
≥ 13 y	65 (30.4)	106 (32.4)	
Body mass index (kg/m^2^)	22.4 (3.3)	23.0 (3.3)	0.04
Current drinking (%)			0.71
No	115 (53.7)	181 (55.4)	
Yes	99 (46.3)	146 (44.7)	
Alcohol flushing status (%)			0.39
Never flushing	107 (50.0)	166 (50.8)	
Former flushing	4 (1.9)	2 (0.6)	
Current flushing	103 (48.1)	159 (48.6)	
Presence of medication history (%)			
Hypertension	50 (23.4)	123 (37.6)	0.0005
Hypercholesterolemia	20 (9.4)	52 (15.9)	0.028
Diabetes mellitus	9 (4.2)	29 (8.9)	0.038
Daily intake^a^			
Total energy (kJ)	8418.6 (2546.8)	8388.6 (3145.4)	0.90
Caffeine (mg/4184 KJ)	153.0 (112.2)	194.6 (139.2)	0.0001
Cholesterol (mg/4184 KJ)	157.8 (60.0)	147.7 (60.9)	0.06
Vitamin E (mg/4184 KJ)	4.2 (1.2)	4.2 (1.2)	0.88
Vitamin B-6 (mg/4184 KJ)	0.6 (0.2)	0.6 (0.2)	0.94
Iron (mg/4184 KJ)	3.8 (0.9)	3.8 (1.0)	0.47
Dietary glycemic index	64.9 (4.7)	65.4 (5.2)	0.27

^a ^Nutrient intake was adjusted for total energy intake using the density method, except for dietary glycemic index.

In crude analyses, neither frequency nor amount of alcohol intake during the peak drinking period was significantly associated with the risk of PD among all subjects (Table 2). After adjustment for several potential confounders including sex, age, region of residence, pack-years of smoking, years of education, body mass index, flushing status, and presence of selected medication history, the association between weekly alcohol intake and PD was strengthened. Compared with non-drinkers, the OR of those who drank ≥219.4 g ethanol per week was 2.09 (95% CI: 1.13-3.91, *P *for trend = 0.03). The confounding was primarily due to smoking, not flushing status (data not shown). This positive relationship disappeared, however, when caffeine, cholesterol, vitamin E, vitamin B6, iron, and the dietary glycemic index were further included as confounders (OR: 1.79, 95% CI: 0.95-3.39, *P *for trend = 0.11). Likewise, no significant associations were observed between frequency or daily ethanol intake and the risk of PD in the fully adjusted model: the adjusted OR of the highest category (≥6 days per week and ≥66.0 g ethanol per day) was 0.96 for the frequency (95% CI: 0.50-1.81, *P *for trend = 0.96) and 1.46 for the daily amount (95% CI: 0.79-2.71, *P *for trend = 0.26).

**Table 2:** ORs for Parkinson's disease in relation to alcohol drinking during "peak" period

**Alcohol drinking during "peak" period **^**a**^	n (%)		Crude OR (95% CI)	**Adjusted OR**^**b**^ (95% CI)	**Adjusted OR**^**c**^ (95% CI)
				
	Cases (N = 214)	Controls (N = 327)			
Frequency					
Non-drinker	115 (53.7)	181 (55.4)	1.00	1.00	1.00
< 6 days per week	63 (29.4)	77 (23.6)	1.29 (0.86-1.93)	1.46 (0.90-2.36)	1.29 (0.78-2.13)
≥ 6 days per week	36 (16.8)	69 (21.1)	0.82 (0.51-1.30)	1.13 (0.61-2.10)	0.96 (0.50-1.81)
*P *for trend			0.70	0.50	0.96
Amount per day (ethanol, g)					
Non-drinker	115 (53.7)	181 (55.4)	1.00	1.00	1.00
0.1-65.9	46 (21.5)	73 (22.3)	0.99 (0.64-1.53)	1.20 (0.72-1.97)	1.07 (0.64-1.80)
≥ 66.0	53 (24.8)	73 (22.3)	1.14 (0.75-1.74)	1.75 (0.97-3.20)	1.46 (0.79-2.71)
*P *for trend			0.58	0.07	0.26
Amount per week (ethanol, g)					
Non-drinker	115 (53.7)	181 (55.4)	1.00	1.00	1.00
0.1-219.3	41 (19.2)	73 (22.3)	0.88 (0.56-1.38)	1.11 (0.67-1.83)	0.98 (0.58-1.65)
≥ 219.4	58 (27.1)	73 (22.3)	1.25 (0.82-1.90)	2.09 (1.13-3.91)	1.79 (0.95-3.39)
*P *for trend			0.38	0.03	0.11

OR, odds ratio; CI, confidence interval.

^a ^Defined as period during which the subject's alcohol consumption was the highest.

^b ^Adjusted for sex, age, region of residence, pack-years of smoking, years of education, body mass index, alcohol flushing status, and presence of medication history for hypertension, hypercholesterolemia, and diabetes.

^c ^Adjusted for sex, age, region of residence, pack-years of smoking, years of education, body mass index, alcohol flushing status, presence of medication history for hypertension, hypercholesterolemia, and diabetes, dietary intake of caffeine, cholesterol, vitamin E, vitamin B6, iron, and dietary glycemic index.

When we assessed daily ethanol intake separately for each type of alcohol, there were no significant associations with PD, except for Japanese sake (Table 3). The OR of the highest category (≥66.0 g ethanol per day) in the fully adjusted model was 2.13 for beer (95% CI: 0.80-5.82, *P *for trend = 0.39), 3.39 for Japanese sake (95% CI: 1.10-11.0, *P *for trend = 0.001), 1.29 for shochu (95% CI: 0.59-2.78, *P *for trend = 0.58), 6.11 for wine (95% CI: 0.67-134, *P *for trend = 0.36) and 2.25 for whisky (95% CI: 0.67-7.83, *P *for trend = 0.06). We could not examine the frequency or weekly amount of alcohol intake for the individual types of alcohol because we did not ask information on drinking frequency separately for each type of alcohol.

**Table 3:** ORs for Parkinson's disease in relation to alcohol drinking amount per day during "peak" period according to types of alcohol

**Alcohol drinking amount per day during "peak" period (ethanol, g) **^**a**^	n (%)		Crude OR (95% CI)	**Adjusted OR**^**b**^ (95% CI)	**Adjusted OR**^**c**^ (95% CI)
				
	Cases (N = 214)	Controls (N = 327)			
Beer					
Non-drinker	127 (59.4)	195 (59.6)	1.00	1.00	1.00
0.1-65.9	75 (35.1)	121 (37.0)	0.95 (0.66-1.37)	1.14 (0.72-1.79)	0.99 (0.61-1.59)
≥ 66.0	12 (5.6)	11 (3.4)	1.68 (0.71-3.97)	2.23 (0.84-5.97)	2.13 (0.80-5.82)
*P *for trend			0.62	0.19	0.39
Japanese sake (rice wine)					
Non-drinker	146 (68.2)	261 (79.8)	1.00	1.00	1.00
0.1-65.9	58 (27.1)	59 (18.0)	1.76 (1.16-2.66)	2.42 (1.44-4.11)	2.27 (1.34-3.89)
≥ 66.0	10 (4.7)	7 (2.1)	2.55 (0.96-7.17)	3.10 (1.04-9.71)	3.39 (1.10-11.0)
*P *for trend			0.002	0.001	0.001
Shochu (a distilled alcoholic beverage made in Japan)					
Non-drinker	180 (84.1)	273 (83.5)	1.00	1.00	1.00
0.1-65.9	18 (8.4)	32 (9.8)	0.85 (0.46-1.55)	1.07 (0.54-2.08)	1.01 (0.50-1.98)
≥ 66.0	16 (7.5)	22 (6.7)	1.10 (0.56-2.15)	1.50 (0.69-3.21)	1.29 (0.59-2.78)
*P *for trend			0.98	0.34	0.58
Wine					
Non-drinker	187 (87.4)	294 (89.9)	1.00	1.00	1.00
0.1-65.9	24 (11.2)	32 (9.8)	1.18 (0.67-2.06)	1.21 (0.66-2.21)	1.06 (0.57-1.95)
≥ 66.0	3 (1.4)	1 (0.3)	4.72 (0.60-95.7)	6.88 (0.75-153)	6.11 (0.67-134)
*P *for trend			0.24	0.18	0.36
Whisky					
Non-drinker	177 (82.7)	283 (86.5)	1.00	1.00	1.00
0.1-65.9	30 (14.0)	38 (11.6)	1.26 (0.75-2.11)	1.62 (0.89-2.94)	1.60 (0.88-2.93)
≥ 66.0	7 (3.3)	6 (1.8)	1.87 (0.61-5.88)	2.72 (0.82-9.34)	2.25 (0.67-7.83)
*P *for trend			0.17	0.03	0.06

OR, odds ratio; CI, confidence interval.

^a ^Defined as period during which the subject's alcohol consumption was the highest.

^b ^Adjusted for sex, age, region of residence, pack-years of smoking, years of education, body mass index, alcohol flushing status, and presence of medication history for hypertension, hypercholesterolemia and diabetes.

^c ^Adjusted for sex, age, region of residence, pack-years of smoking, years of education, body mass index, alcohol flushing status, presence of medication history for hypertension, hypercholesterolemia and diabetes, dietary intake of caffeine, cholesterol, vitamin E, vitamin B6, iron, and dietary glycemic index.

In the stratified analysis according to flushing status, former and current flushers were combined due to small numbers of former flushers (Table 4). We did not find any significant interactions between alcohol drinking and flushing status with regard to the risk of PD (*P *for interaction = 0.22, 0.37, and 0.18 for the highest category of frequency, daily amount, and weekly amount of alcohol intake, respectively). With respect to the separate types of alcohol, no interactions with flashing status were observed.

**Table 4:** ORs for Parkinson's disease in relation to alcohol drinking during "peak" period by flushing status

**Alcohol drinking during "peak" period **^**a**^	n (%)		Crude OR (95% CI)	**Adjusted OR**^**b **^ (95% CI)	**Adjusted OR**^**c **^ (95% CI)
***Never flushing***	***Cases*** ***(N = 107)***	***Controls*** ***(N = 166)***			
Frequency					
Non-drinker	79 (73.8)	127 (76.5)	1.00	1.00	1.00
< 6 days per week	23 (21.5)	24 (14.5)	1.54 (0.81-2.92)	1.65 (0.80-3.44)	1.32 (0.60-2.93)
≥ 6 days per week	5 (4.7)	15 (9.0)	0.54 (0.17-1.44)	0.78 (0.22-2.54)	0.61 (0.17-1.99)
*P *for trend			0.82	0.67	0.77
Amount per day (ethanol, g)					
Non-drinker	79 (73.8)	127 (76.5)	1.00	1.00	1.00
0.1-65.9	17 (15.9)	21 (12.7)	1.30 (0.64-2.61)	1.33 (0.61-2.87)	1.07 (0.47-2.43)
≥ 66.0	11 (10.3)	18 (10.8)	0.98 (0.43-2.16)	1.54 (0.54-4.39)	1.11 (0.37-3.31)
*P *for trend			0.80	0.32	0.82
Amount per week (ethanol, g)					
Non-drinker	79 (73.8)	127 (76.5)	1.00	1.00	1.00
0.1-219.3	19 (17.8)	23 (13.9)	1.33 (0.68-2.59)	1.46 (0.69-3.09)	1.18 (0.53-2.63)
≥ 219.4	9 (8.4)	16 (9.6)	0.90 (0.37-2.11)	1.24 (0.42-3.60)	0.89 (0.29-2.66)
*P *for trend			0.85	0.44	0.99
***Former/current flushing***	***Cases*** ***(N = 107)***	***Controls*** ***(N = 161)***			
Frequency					
Non-drinker	36 (33.6)	54 (33.5)	1.00	1.00	1.00
< 6 days per week	40 (37.4)	53 (32.9)	1.13 (0.63-2.04)	1.12 (0.56-2.26)	0.97 (0.47-2.02)
≥ 6 days per week	31 (29.0)	54 (33.5)	0.86 (0.47-1.59)	0.98 (0.43-2.26)	0.84 (0.35-2.00)
*P *for trend			0.64	0.96	0.68
Amount per day (ethanol, g)					
Non-drinker	36 (33.6)	54 (33.5)	1.00	1.00	1.00
0.1-65.9	29 (27.1)	52 (32.3)	0.84 (0.45-1.55)	0.97 (0.48-1.97)	0.84 (0.40-1.77)
≥ 66.0	42 (39.3)	55 (34.2)	1.15 (0.64-2.06)	1.38 (0.61-3.17)	1.16 (0.49-2.74)
*P *for trend			0.63	0.44	0.71
Amount per week (ethanol, g)					
Non-drinker	36 (33.6)	54 (33.5)	1.00	1.00	1.00
0.1-219.3	22 (20.6)	50 (31.1)	0.66 (0.34-1.26)	0.79 (0.38-1.64)	0.66 (0.30-1.43)
≥ 219.4	49 (45.8)	57 (35.4)	1.29 (0.73-2.29)	2.06 (0.88-4.96)	1.82 (0.75-4.53)
*P *for trend			0.33	0.13	0.23

OR, odds ratio; CI, confidence interval.

^a ^Defined as period during which the subject's alcohol consumption was the highest.

^b ^Adjusted for sex, age, region of residence, pack-years of smoking, years of education, body mass index, and presence of medication history for hypertension, hypercholesterolemia, and diabetes.

^c ^Adjusted for sex, age, region of residence, pack-years of smoking, years of education, body mass index, presence of medication history for hypertension, hypercholesterolemia, and diabetes, dietary intake of caffeine, cholesterol, vitamin E, vitamin B6, iron, and dietary glycemic index.

## Discussion

In this study, alcohol intake during the peak drinking period was not associated with PD when ethanol intake was combined from all types of alcohol. There was no significant interaction of alcohol drinking with flushing status in relation to PD risk. Similarly to the present study, a substantial body of previous case-control studies failed to find significant associations between alcohol and PD. In each study, alcohol as an exposure variable was assessed differently, including as a binary category of "ever vs. never" [5-8,12,13], an average amount [12], a cumulative amount [10], or amount per week based on typical consumption patterns during most of the subject's adult life [9]. A large prospective cohort study, including two cohorts from the Nurses' Health Study and Health Professionals' Study, found no significant relationship between average alcohol intake at baseline and subsequent PD incidence [11].

Our finding is not in agreement with previous studies that observed significant inverse associations between alcohol intake and PD [1-4]. A meta-analysis reported a reduced risk of PD among drinkers compared with non-drinkers: the pooled OR (95% CI) was 0.81(0.70-0.92) or 0.73 (0.57-0.92) when 13 case-control studies or 4 cohort studies, respectively, were included [14]. Several epidemiologic studies found that beer [11,28,29], spirits [28], wine [29] and liquor [2,29], but not ethanol intake [11,28], were inversely associated with PD. The high content of urate in beer or niacin in alcoholic beverages were reported as plausible protective agents [30,31]. Another hypothesis is that PD patients have a premorbid personality to avoid alcohol drinking. Two previous case-control studies showed that PD patients were significantly less likely to have been diagnosed with alcoholism or to have a previous history of alcohol use disorder [8,13].

When we examined daily ethanol intake separately for each type of alcohol, only Japanese sake was positively associated with PD. To our knowledge, there has been no report that Japanese sake increased the risk of PD. Although our finding could be due to chance, it has been shown that relationships between alcohol intake and PD varied according to different kinds of alcohol [11,28,29].

*In vitro *studies showed that aldehydes might react with dopamine. Ethanol enhanced the toxicity of 6-hydroxydopamin (6-OHDA) when ethanol and 6-OHDA were simultaneously applied to cultured cells [20]. In an attempt to create a mouse version of the rat model of PD, developed using a synthetic proteasome inhibitor (PSI), decreased levels of nigrostriatal dopamine were observed both in the mice treated with PSI in an ethanol-vehicle and in control mice with ethanol-vehicle alone [21]. By contrast, it was reported that ADHs, and not ALDHs, play important roles in the synthesis of retinoic acid, which may influence the proper development and maintenance of the dopaminergic system [32]. These inconsistent findings may explain the lack of significant interaction we observed between alcohol drinking and flushing status in relation to PD risk.

A strength of the present study is that cases were identified using strict diagnostic criteria, minimizing disease misclassification as much as possible. We also designed our study with extensive data collection, based on comprehensive literature review, allowing us to adjust for several potential confounders, including dietary factors. However, residual confounding cannot be ruled out. In contrast to previous studies that looked at average drinking habits, we examined subjects' peak drinking status as exposure variables. Some PD patients experience several gastrointestinal symptoms as non-motor manifestations. It was shown that constipation, occurring as early as 20 or more years before the onset of motor symptoms, was associated with an increased risk of PD [33]. Given that some gastrointestinal symptoms such as constipation affect alcohol drinking status even before the onset of disease, average or cumulative drinking amounts might be decreased in PD patients compared with healthy subjects. Thus the peak drinking status may be another reasonable indicator to avoid underestimation of alcohol consumption among PD patients.

Our study was limited by our failure to collect information on peak drinking from former drinkers (approximately 10% of the subjects initially recruited for the present study). A comparison of the 541 subjects included in the final analysis with 69 former drinkers revealed that former drinkers were more likely to be male (63.8 vs. 34.6%, *P *<0.0001), older (mean age: 70.2 vs. 67.0, *P *= 0.003), smokers (those with ≥30.0 pack-years: 36.2 vs. 15.3%, *P *<0.0001), more educated (those with ≥13 years: 34.8 vs. 31.6%, *P *= 0.006), and have a medication history for diabetes (15.9 vs. 7.0%, *P *= 0.01). We previously revealed an inverse association between smoking habits or medication history for diabetes and PD in this study population [22,23]. By contrast, ageing is known to be strong positive risk factor for PD; therefore, selection bias might not be negligible, although we cannot precisely speculate on either the direction or the magnitude of the bias.

Another possible limitation is that our controls were not fully representative of the population from which our cases arose, as they were selected from only 3 of the 11 collaborating hospitals where cases were recruited. The results of a sensitivity analysis restricted to cases who were recruited from the three hospitals associated with control recruitment (n = 130) were similar to those in the overall analysis: the adjusted OR in the highest category was 0.78 (95% CI: 0.36-1.63, *P *for trend = 0.59) for frequency, 1.17 (95% CI: 0.58-2.36, *P *for trend = 0.71) for daily amount, and 1.66 (95% CI: 0.81-3.42, *P *for trend = 0.26) for weekly amount. Furthermore, cases in the present study were prevalent rather than incident cases. When we conducted a sensitivity analysis confined to cases less than 3 years from onset (n = 88), interpretation of our results was not markedly changed. The adjusted OR in the highest category was 0.60 (95% CI: 0.24-1.42, *P *for trend = 0.32) for frequency, 1.28 (95% CI: 0.58-2.82, *P *for trend = 0.69) for daily amount, and 1.50 (95% CI: 0.67-3.36, *P *for trend = 0.51) for weekly amount. We were also concerned that medication history might affect alcohol drinking habits, so we performed a sensitivity analysis among subjects without medication history for hypertension, hypercholesterolemia, or diabetes (152 cases and 173 controls). The associations between alcohol intake and PD were not considerably altered. The adjusted OR in the highest category was 0.91 (95% CI: 0.41-2.00, *P *for trend = 0.94) for frequency, 1.34 (95% CI: 0.62-2.93, *P *for trend = 0.47) for daily amount, and 1.61 (95% CI: 0.75-3.50, *P *for trend = 0.27) for weekly amount. Thus, the possible influence of medication history on alcohol intake was, if any, likely to be minimal. Finally, all the information in this study relied on self-reports rather than on interviews by trained investigators, and information on alcohol intake and non-dietary factors was collected via a non-validated questionnaire, which could affect the present results.

## Conclusions

We did not find significant associations between alcohol intake and PD, except for the daily amount of Japanese sake. Effect modifications by alcohol flushing status were not observed. The present study, however, is the first to assess the effects of alcohol on PD risk using peak drinking status as an exposure variable and alcohol flushing status as a potential confounder. To better understand the role of alcohol drinking in the pathogenesis of PD, it may be helpful to take into consideration many aspects of alcohol.

## Competing interests

The authors declare that they have no competing interests.

## Authors' contributions

WF contributed to study design, data collection, data management, statistical analysis, data interpretation, and manuscript writing. YM contributed to study design, data collection, overall management, data interpretation, and manuscript editing. SS contributed to design of the dietary study. KT and CK contributed to study design, data collection, and data management. YT, TY, TO, TM, NK, NS, and HF contributed to outcome definition and case recruitment. YH and MN contributed to conception of the design and execution of the study. Authors listed in the Appendix contributed to case or control subject recruitment. All authors provided comments on the drafts and have read and approved the final version.

## Appendix

Other members of the Fukuoka Kinki Parkinson's Disease Study Group are as follow: Yasuhiko Baba and Tomonori Kobayashi (Department of Neurology, Faculty of Medicine, Fukuoka University); Hideyuki Sawada, Eiji Mizuta, and Nagako Murase (Clinical Research Institute and Department of Neurology, Utano National Hospital); Tsuyoshi Tsutada and Hiroyuki Shimada (Department of Geriatrics and Neurology, Osaka City University Graduate School of Medicine); Jun-ichi Kira (Department of Neurology, Neurological Institute, Graduate School of Medical Sciences, Kyushu University); Tameko Kihira and Tomoyoshi Kondo (Department of Neurology, Wakayama Medical University); Hidekazu Tomimoto (Department of Neurology, Kyoto University Graduate School of Medicine); Takayuki Taniwaki (Division of Respirology, Neurology, and Rheumatology, Department of Medicine, Kurume University School of Medicine); Hiroshi Sugiyama and Sonoyo Yoshida (Department of Neurology, Minami-Kyoto National Hospital); Harutoshi Fujimura and Tomoko Saito (Department of Neurology, Toneyama National Hospital); Kyoko Saida and Junko Fujitake (Department of Neurology, Kyoto City Hospital); Naoki Fujii (Department of Neurology, Neuro-Muscular Center, National Omuta Hospital); Masatoshi Naito and Jun Arimizu (Department of Orthopaedic Surgery, Faculty of Medicine, Fukuoka University); Takashi Nakagawa, Hirofumi Harada, and Takayuki Sueta (Department of Otorhinolaryngology, Faculty of Medicine, Fukuoka University); Toshihiro Kikuta and George Umemoto (Department of Oral and Maxillofacial Surgery, Faculty of Medicine, Fukuoka University); Eiichi Uchio and Hironori Migita (Department of Ophthalmology, Faculty of Medicine, Fukuoka University); Kenichi Kazuki, Yoichi Ito, and Hiroyoshi Iwaki (Department of Orthopaedic Surgery, Osaka City University Graduate School of Medicine); Kunihiko Siraki and Shinsuke Ataka (Department of Ophthalmology and Visual Sciences, Osaka City University Graduate School of Medicine); Hideo Yaname and Rie Tochino (Department of Otolaryngology and Head and Neck Surgery, Osaka City University Graduate School of Medicine); Teruichi Harada (Plastic and Reconstructive Surgery, Osaka City University Graduate School of Medicine); Yasushi Iwashita, Motoyuki Shimizu, Kenji Seki, and Keiji Ando (Department of Orthopedic Surgery, Utano National Hospital).

## Pre-publication history

The pre-publication history for this paper can be accessed here:

http://www.biomedcentral.com/1471-2377/10/111/prepub
